# Optimized tacrolimus dosing strategy in kidney transplant recipients receiving nirmatrelvir-ritonavir for COVID-19

**DOI:** 10.1371/journal.pone.0309875

**Published:** 2025-05-30

**Authors:** Han Yan, Shanbiao Hu, Hedong Zhang, Yangang Zhou, Rao Fu, Ping Xu, Hualin Cai, Xi Li, Gongbin Lan

**Affiliations:** 1 Department of Pharmacy, The Second Xiangya Hospital, Central South University, Changsha, Hunan, P.R. China; 2 Institute of Clinical Pharmacy, Central South University, Changsha, China; 3 Department of Kidney Transplantation, The Second Xiangya Hospital, Central South University, Changsha, Hunan, P.R. China; 4 Department of Clinical Pharmacology, Xiangya Hospital, Central South University, Changsha, Hunan, P.R. China; 5 Institute of Clinical Pharmacology, Central South University, Hunan Key Laboratory of Pharmacogenetics, Changsha, Hunan, P.R. China; University of Colombo Faculty of Medicine, SRI LANKA

## Abstract

Kidney transplantation recipients (KTRs) represent a vulnerable population for COVID-19 infection and severe disease. Nirmatrelvir-ritonavir has demonstrated efficacy in treating COVID-19 among KTRs, and interacts with tacrolimus leading to a precipitous increase in tacrolimus blood levels when co-administered, which may potentially result in toxicity. To explore a safe strategy for the combination of nirmatrelvir-ritonavir and tacrolimus, we established a new administration strategy to restore tacrolimus after the discontinuation of nirmatrelvir-ritonavir and conducted a real-world retrospective observational cohort study to evaluate its clinical efficacy. In the experimental group, tacrolimus was initiated at 20–25% of the baseline dose 48 hours after the discontinuation of nirmatrelvir-ritonavir, with daily increments of 20–25% until the baseline dose was restored. The patients who did not follow the experimental protocol were included in the control group. Results showed that withholding tacrolimus 12 hours before starting nirmatrelvir-ritonavir maintained tacrolimus blood levels above 83% of the baseline throughout the nirmatrelvir-ritonavir treatment period. Compared with the control group, the experimental group achieved target trough concentrations of tacrolimus more quickly and maintained a higher proportion within the therapeutic range (*p* = 0.029), and had significantly lower rates of adverse events (*p = *0.002, OR = 0.308, 95%CI:0.136–0.695). This study provides a safe and effective pharmacological strategy for KTRs infected with COVID-19, allowing the safe co-administration of nirmatrelvir-ritonavir and tacrolimus.

## Introduction

Since the spring of 2020, the world has been experiencing a global COVID-19 pandemic, with the Omicron variant now being the predominant strain spreading widely in communities across various countries [1]. Solid organ transplant recipients (SOTRs) are more susceptible to COVID-19 due to long-term immunosuppressant use, which leads to a decline in immune function and a weaker response to vaccines [2]. Consequently, the virus removal efficiency decreased in SOTRs, and they are at a higher risk of developing severe COVID or death compared to the general population. Multiple studies have demonstrated that SOTR infected with COVID-19 exhibit a significantly elevated mortality rate [3–8]. Therefore, it is essential for SOTRs to seek prompt treatment when infected with COVID-19 to prevent progression to severe disease [9].

Paxlovid, a medication manufactured by Pfizer for the treatment of COVID-19, consists of nirmatrelvir and ritonavir. Nirmatrelvir is a novel inhibitor targeting the 3Clpro of SARS-CoV-2 and ritonavir is a potent inhibitor of cytochrome P450 3A, which can reduce the metabolism of nirmatrelvir and elevate its serum levels [10,11]. A phase 2/3 clinical trial indicated that nirmatrelvir-ritonavir reduced the risk of symptomatic COVID-19 progressing to severe disease by 89% compared to the control group [12].

Ritonavir is a potent inhibitor of the CYP3A enzyme system. Thus, its interactions with CYP3A-dependent medications may lead to a significant increase in the area under the curve (AUC). Tacrolimus, which has a narrow therapeutic window, is commonly used for maintenance anti-rejection therapy after renal transplantation, and any concentration below or exceeding the recommended range could lead to insufficient immunosuppression or toxicity [13,14]. When exposed to ritonavir, the plasma level of tacrolimus will rapidly and significantly increase by approximately 50 times [15]. Additionally, after the discontinuation of nirmatrelvir-ritonavir, the inhibitory effect on CYP3A enzymes can last for several days, and resuming the original dose of tacrolimus instantly would result in a rapid rise in its concentration, potentially causing toxic reactions and increasing the complexity of treatment [16,17].

Several studies have explored the rational use of nirmatrelvir-ritonavir and tacrolimus in SOTRs. The Expert Consensus on the diagnosis and treatment of novel coronavirus infection in SOTRs (2023, China) suggests adjusting the tacrolimus dose by administering a 1/2 daily dose 24 hours after the completion of the nirmatrelvir-ritonavir course, followed by a 3/4 daily dose at 48 hours, and resuming the original dose 72 hours later. However, when this regimen was applied to our patients, their tacrolimus levels increased significantly, which led to COVID - 19 recurrence. Lange *et al.* recommended monitoring tacrolimus levels on the 6th or 7th day of nirmatrelvir-ritonavir initiation and then repeating the monitoring every 2–4 days [18]. Based on whether the detected concentration was within the target concentration range, the tacrolimus dose could be held or restarted with 25% to 75% of the baseline dose [18]. However, this frequent monitoring scheme is not suitable for outpatients. Additionally, the activity of the CYP450 enzyme gradually recovers after the discontinuation of nirmatrelvir-ritonavir [19], and adjusting the dose only every 2–4 days might result in suboptimal concentrations, potentially increasing the risk of rejection. Therefore, it is urgently necessary to conduct further research to determine the optimal timing and dosing regimen for the concurrent use of nirmatrelvir-ritonavir and tacrolimus in renal transplant patients [20–23]. Ensuring the effective treatment of COVID-19 and drug safety is paramount for these patients.

To explore a safe strategy for combining nirmatrelvir-ritonavir and tacrolimus, a real-world retrospective observational cohort study on kidney transplantation recipients (KTRs) with COVID-19 was performed. We developed a new administration strategy for restoring tacrolimus after the discontinuation of nirmatrelvir-ritonavir. Patients who adopted this new strategy were enrolled in the experimental group, and those who did not use this new strategy were assigned to the control group. Subsequently, we compared the clinical outcomes of the two groups to determine whether the new strategy could reduce the risk of adverse events.

## Methods

### Patient selection

**Fig 1:**
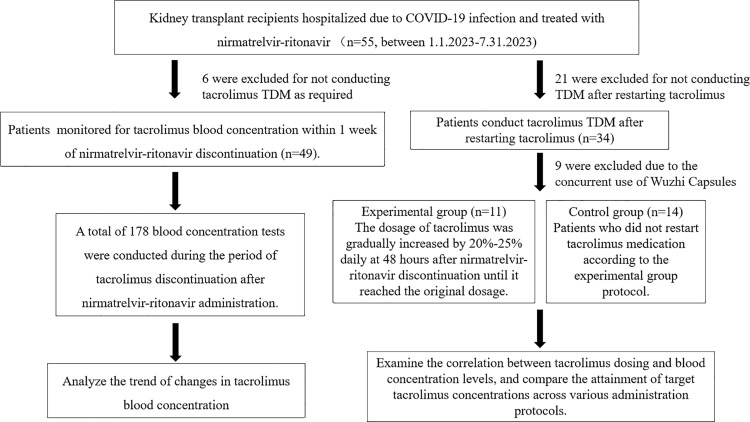
Flow chart of the study.

This was a real-world retrospective observational cohort study. The research flowchart is presented in Fig 1. The data for this study were obtained from the Transplantation Department of the Second Xiangya Hospital, Central South University. We collected clinical data from KTRs infected with COVID-19 between January 1, 2023, and July 31, 2023, along with six months follow-up data post-infection. The study data were accessed from January 25, 2024, to February 24, 2024. All participants had written the informed consent form during data collection. The inclusion criteria were as follows: (i) post-kidney transplantation; (ii) admitted to the Second Xiangya Hospital; (iii) diagnosis of COVID-19 infection with a CT value <30/30 for SARS-CoV-2 nucleic acid; (iv) use tacrolimus for anti-rejection regimen; (v) received nirmatrelvir-ritonavir (Paxlovid, Pfizer) treatment; and (vi) age > 18 years and weight >40 kg. Patients who were pregnant or breastfeeding, HIV-infected, did not complete the nirmatrelvir-ritonavir treatment course, lacked accessible clinical information, or did not write an informed consent were excluded.

According to the inclusion and exclusion criteria, a total of 55 patients were enrolled. Our study was divided into two parts. The first part was to explore the changes in blood concentration of tacrolimus after its discontinuation, during and after nirmatrelvir-ritonavir treatment. Among the 55 patients, 6 were excluded for not conducting tacrolimus TDM as required. In the remaining 49 enrolled patients, all of the included tacrolimus blood concentrations were detected before the initiation of tacrolimus. For patients who had already been taking tacrolimus, the subsequent concentration measurement data were not included in this part of the analysis.

The second part of the study aimed to explore the tacrolimus dosing regimen after the discontinuation of nirmatrelvir-ritonavir. Of the 55 patients, 21 were excluded for not conducting concentration measurement after restarting tacrolimus. Additionally, 9 were excluded due to the concurrent use of Wuzhi Capsules. Consequently, a total of 25 patients were included in this part of the study, who had their tacrolimus blood concentration monitored in the hospital after restarting tacrolimus following the discontinuation of nirmatrelvir-ritonavir. For details, see Fig 1.

### Therapeutic regimen

**Fig 2:**
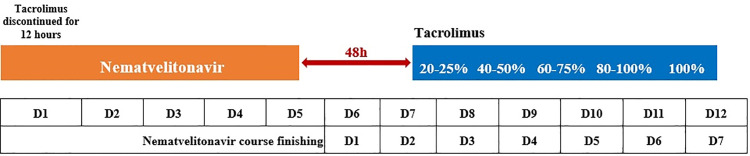
The regimen of commencing tacrolimus after nirmatrelvir-ritonavir withdrawal in the experimental group.

All patients hold tacrolimus 12 hours before starting nirmatrelvir-ritonavir and tacrolimus levels were measured on day 0 (D0). In the experimental group, patients started to receive 20–25% of the baseline dose of tacrolimus 48 hours (D7) after nirmatrelvir-ritonavir withdrawal. On D8, they received 40–50% of the baseline dose. On D9 60–75%, on D10 80–100%, and 100% on D11 (day 7 after nirmatrelvir-ritonavir suspension) [17,19,23], as shown in Fig 2.

### Samples grouping

This was a real-world research. Patients who followed the dosing schedule described in Fig 2 were assigned to the experimental group. All 11 patients in the experimental group resumed the original tacrolimus dosage on either the sixth or seventh day after discontinuing nirmatrelvir-ritonavir. The remaining 14 patients not on this dosing schedule were classified as the control group, including those who had not resumed the original tacrolimus dosage by the seventh day after stopping nirmatrelvir-ritonavir or had started tacrolimus within 48 hours, as well as those who resumed the original dosage prior to the sixth day. Tacrolimus levels were monitored every 2–3 days during hospitalization, and regular outpatient monitoring of tacrolimus levels and renal function was conducted after discharge.

Information on medication treatment response, including symptom improvement and adverse reactions, was obtained through bedside inquiry or telephone follow-up. Patient outcomes and laboratory test results were retrieved from the medical record system. This study was approved by the ethics committee of the Second Xiangya Hospital of Central South University (LYEC2024–0016).

### Microbiology

Nasopharyngeal swab testing for SARS-CoV-2 was conducted by using real-time fluorescent quantitative RT-PCR technology, targeting the ORF1ab gene and the gene encoding the nucleocapsid protein N for dual-target detection.

### Tacrolimus level monitoring

The study included 49 patients with a total of 219 tacrolimus trough concentrations, 41 of which were post-dose data. Tacrolimus level monitoring was performed by using Chemiluminescent Microparticle Immunoassay (CMIA) technology on the ARCHITECT i system to quantify FK506 in human whole blood. The test kits were obtained from Abbott Laboratories (United).

### Main outcomes

The primary outcome of this study was the proportion of tacrolimus concentrations within the target range, defined as the number of tacrolimus measurements within 20% of the baseline concentration divided by the total number of concentration measurements. For example, during Days 1–2, the experimental group collected data from five concentration measurements. Among them three measurements were within 20% of the baseline concentration, one was above 20%, and one was below 20%. Thus, the proportion of concentrations within the target range for the experimental group on Days 1–2 was 3/5. The control group collected data from eight concentration measurements, with only one measurement was within 20% of the baseline concentration, so the proportion was 1/8. To facilitate comparison between different samples, we also calculated the relative concentration of tacrolimus, which refers to the proportion of tacrolimus concentration in relation to the baseline concentration. Another outcome was the cumulative compliance ratio of patients achieving the target concentration, which was defined as the percentage of patients who achieved the target concentration at least once during the follow-up period.

### Statistical analysis

In this study, all data were analyzed and plotted by using Origin 2020b (Educational Version). The cumulative survival curve was plotted using the Kaplan-Meier method, and the log-rank test was utilized to assess significant differences in cumulative risk. The Cox regression analysis was used for calculating the HR and its 95% confidence interval (95% CI). Independent sample t-test and Fisher exact test were used to compare significant differences between numerical and categorical variables, respectively. For multi group comparisons, one-way ANOVA test was utilized. All tests were two-tailed, and *p* < 0.05 was considered statistically significant.

## Results

### Baseline characteristics

A total of 49 hospitalized kidney transplantation patients meeting the criteria for COVID-19 infection admitted to the Second Xiangya Hospital were enrolled. The baseline characteristics of the cohort are presented in Table 1. In brief, the median age was 47 years (ranging from 18–65), and 30 patients (61.2%) were male. The median time from transplantation to COVID-19 diagnosis was 44 months (ranging from 0–240). Among these patients 8 (16.3%) with diabetes, 21 (42.9%) with hypertension, 4 (8.2%) with osteoporosis, and 21 (42.9%) with anemia. Immunosuppressive treatment mainly included a combination of tacrolimus (100%), mycophenolate mofetil (93.9%), and steroids (100%). 19 patients (38.8%) received medium-dose steroid anti-inflammatory treatment. Only 9 patients (18.4%) and 7 patients (14.3%) were vaccinated with three doses and two doses, respectively. None of the patients had a history of Sars-CoV-2 infection, and the median time from symptom onset to hospital admission was 4 days (ranging from 0–30 days). The symptoms after infection included fever (73.4%), cough (67.3%), fatigue (40.8%), muscle and throat pain (38.8%), and chest tightness and shortness of breath (28.6%). All patients completed 5 days of nirmatrelvir-ritonavir therapy, with dosage adjustments as follows: for GFR > 30ml/min/1.73m^2 given 300/100mg q12h; for GFR between 10–30ml/min/1.73m^2 given 150/100mg q12h; for GFR < 10ml/min/1.73m^2 given 150/100mg qd.

**Table 1:** Baseline characteristics of the cohort.

Variable	Population N = 49
Age, years, median (ranges)	47 (18-65)
Male sex, n(%)	30 (61.2)
Time from txp to COVID-19, months (ranges)	44 (0-240)
Serum creatinine before, mL/(min*1.73m^2^)	40.7 (7.7-99.3)
Serum creatinine after,(min*1.73m^2^)	40.8 (8.6-135.1)
Time of onset of symptoms to admission, d (ranges)	4 (0-30)
Symptom, n(%)	
Fever	36 (73.4)
chest distress and shortness of breath	14 (28.6)
Cough	33 (67.3)
gastrointestinal symptoms	10 (20.4)
fatigue	20 (40.8)
ache	19 (38.8)
Complicating disease, n(%)	
Diabetes	8(16.3)
Hypertension	21(42.9)
Osteoporosis	4(8.2)
Anemia	21(42.9)
Concomitant drug-drug interactions	
Azole antifungals	3 (6.1)
HMG-CoA reductase inhibitors	4 (8.2)
Hypotensive drugs (CCB)	16 (32.7)
Repaglinide	3 (6.1)
Immunosuppression of admission, n(%)	
Tacrolimus	49 (100.0)
Mycophenolate mofetil	46 (93.9)
Mizoribine	3 (6.1)
Steroids	49 (100.0)
Nirmatrelvir-ritonavir maintenance dose, n (%)	
300/100 mg q12h	29 (59.2)
150/100 mg q12h	19 (38.8)
150/100mg qd	1 (2.0)
Moderate doses of corticosteroids	19 (38.8)
Prior anti-SARS-CoV-2 vaccine, n(%)	
3 doses	9(18.4)
2 doses	7(14.3)
0 dose	33(67.3)

### Clinical efficacy and adverse drug reaction of nirmatrelvir-ritonavir

**Fig 3:**
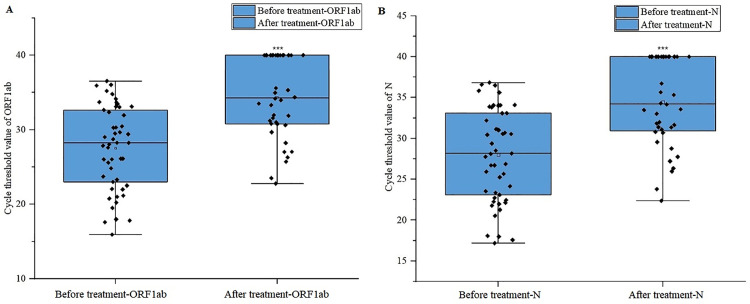
Changes in the SARS-CoV-2 Ct value in patients before and after nirmatrelvir-ritonavir administration. A: ORF1ab gene. B: The gene encoding the nucleocapsid protein N. *** represent p ＜ 0.001.

All 49 patients infected with COVID-19 recovered after being treated with nirmatrelvir-ritonavir. Sixteen patients tested negative for COVID-19 nucleic acid after completing the 5-day treatment course, while the remaining patients either turned antigen negative or had their symptoms disappear within two weeks after discharge. Additionally, two patients with COVID-19 experienced a rebound after one course of nirmatrelvir-ritonavir treatment, and two patients received two courses of nirmatrelvir-ritonavir treatment due to unresolved symptoms. After treatment, the SARS-CoV-2 Ct value increased significantly (*p* < 0.001), indicating a reduction in the viral load (Fig 3). During treatment, 40 patients (81.6%) experienced symptoms such as a bitter taste and altered taste sensation, 13 patients (26.5%) experienced gastrointestinal discomfort, 2 patients (4.1%) experienced dizziness, and 1 patient (2.0%) had itchy hands. These adverse reactions were alleviated after the discontinuation of medication.

### Tacrolimus concentration changes during nirmatrelvir-ritonavir use

**Fig 4:**
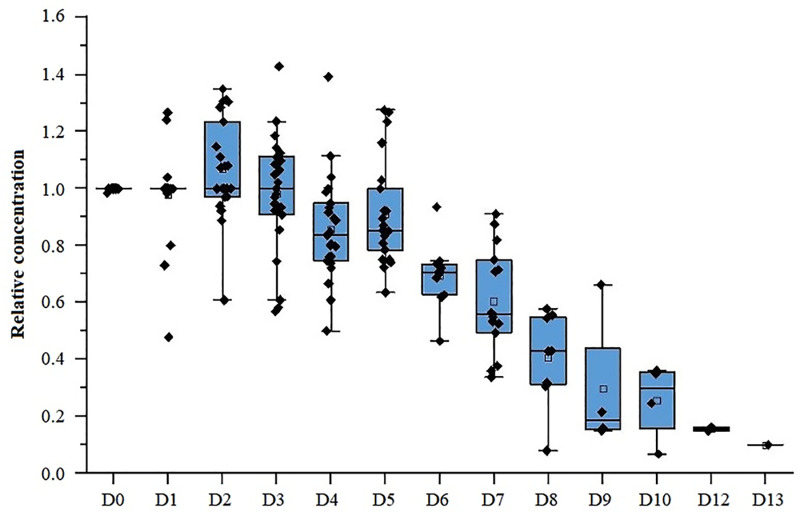
Changes of residual blood concentration of tacrolimus during and after nirmatrelvir-ritonavir use. The TDM data presented in this figure was exclusively collected during the periods when the subjects were not administering tacrolimus due to other reasons such as secondary severe infection or unrelieved symptoms. There is no TDM data on day 11.

All 49 patients discontinued tacrolimus 12 hours before starting nirmatrelvir-ritonavir treatment, and the trough concentration of tacrolimus measured before medication was used as the baseline concentration. Tacrolimus concentrations were monitored every 2–3 days, and 219 blood drug concentration measurements were collected. After excluding 41 post-dose data, 178 TDM data during the tacrolimus discontinuation period were obtained in this study. The results showed that despite the withdrawal of tacrolimus, its concentration could be maintained at a relatively high level during the administration of nirmatrelvir-ritonavir, with D1-D5 trough concentrations of 98%, 107%, 96%, 83%, and 89% of the baseline concentration, respectively. On D6 and D7, tacrolimus concentrations could still be maintained at 69% and 60%, respectively. However on D8 and thereafter, its blood drug concentration dropped significantly to lower levels of 47%, 30%, 26%, None, 16%, and 10%, respectively (Fig 4). Therefore, within 48 hours of ritonavir discontinuation, tacrolimus concentration could still be maintained at a relatively high level.

### Exploration of tacrolimus dosing regimen after nirmatrelvir-ritonavir discontinuation

**Fig 5:**
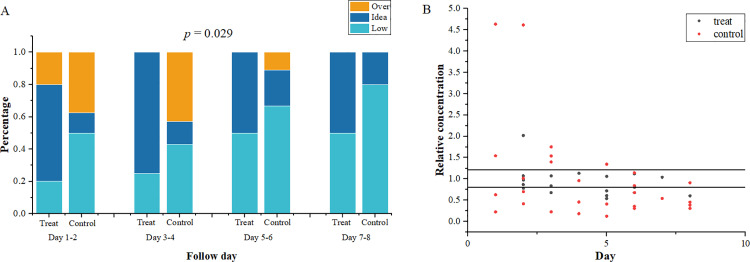
The ratio in the target concentration range of tacrolimus between experimental group and control. A: Ratio of reaching the target concentration range in every two days. B: Scatter plot of relative concentration of tacrolimus. The relative concentration of tacrolimus means the proportion of tacrolimus concentration relative to the baseline concentration.

**Fig 6:**
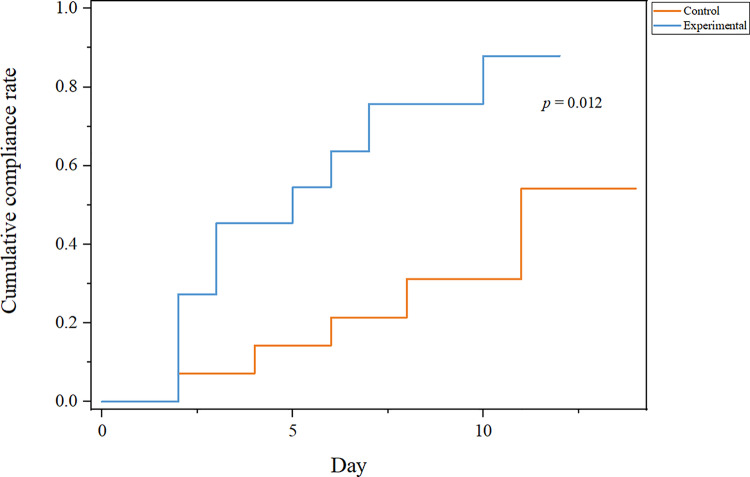
The cumulative compliance ratio curve of tacrolimus between experimental group and control. The cumulative compliance ratio is defined as the percentage of patients who achieved the target concentration at least once during the follow-up period.

This part of the study included 34 patients who resumed tacrolimus treatment after the end of nirmatrelvir-ritonavir treatment and underwent TDM monitoring within 10 days at the Second Xiangya Hospital. After excluding 9 samples who used Wuzhi Capsules (CYP3A enzyme inhibitors, which could increase the oral bioavailability of tacrolimus and increase its blood concentration) together [24], a total of 25 patients were included in this part of study. These 25 patients were divided into two groups: 11 in the experimental group and 14 in the control group, with a total of 69 FK506 concentration tests conducted. Concentration compliance was defined as fluctuations within 20% of the baseline concentration. Due to limited data, concentrations measured every two days were grouped for analysis. The ratio of tacrolimus within the target concentration range in the experimental group was significantly higher than that in the control group (*p* = 0.029) (Fig 5A), and the cumulative compliance ratio analysis showed that the experimental group patients reached the target range faster and in a higher proportion (72.7% vs 35.7%, *p* = 0.012, HR = 3.652, 95%CI:2.542–4.762) (Fig 6). Additionally, concentrations in the experimental group that were outside the target range were still close to the target concentration, whereas those in the control group deviated further from the target range (Fig 5B).

In addition, two patients in the control group started taking 80%, 45% of the basic dose of tacrolimus within 12 hours after the withdrawal of nirmatrelvir-ritonavir, followed by 40% and 100% the next day. Then their concentration significantly increased to more than 4.64 and 4.62 times of the baseline concentration, with 5.5 ng/ml rising to 26 ng/ml, and 6.5 ng/ml rising to more than 30ng/ml. Another patient took 22% of the baseline dose of tacrolimus for two consecutive days after nirmatrelvir-ritonavir discontinuation for 24 hours, and the concentration increased to 2.02 times of the baseline concentration. However, for patients who did not resume tacrolimus 48 hours after nirmatrelvir-ritonavir withdrawal, their concentration was significantly lower than the baseline concentration (Fig 4). Patients who received insufficient doses compared to the experimental group also had significantly lower concentrations than the baseline concentration.

### Events occurring within 6 months after COVID-19 recovery

In this part of the study, two patients who experienced twice COVID-19 infections each received two courses of nirmatrelvir-ritonavir treatment, respectively. Consequently, the number of patients in the control and treat group was 13 and 10, respectively. Among the 13 patients in the control group, 3 were hospitalized due to creatinine elevation within 6 months, 4 had COVID-19 rebound or delayed conversion to negative, 2 experienced opportunistic infections, one with Pneumocystis jiroveci pneumonia (PJP) and the other with Cytomegalovirus (CMV), and one patient died from severe pneumonia triggered by herpes virus infection. The event rate within 6 months in the control group was 69.2%, while none of the patients in the experimental group experienced the aforementioned events. The incidence of events within 6 months after nirmatrelvir-ritonavir treatment for COVID-19 was significantly lower in the experimental group than in the control group (69.2% vs 0%, *p = *0.002, OR = 0.308, 95%CI:0.136–0.695) (Table 2).

**Table 2:** Events occurred within 6 months after treatment of COVID-19 infection with nirmatrelvir-ritonavir.

Events	Control (N = 13)	Experimental (N = 10)	*p* value
Admitted with Creatinine elevation, n(%)	3(23.1)	0(0)	0.229
COVID-19 rebounded or negative conversion time prolonged, n(%)	4(30.8)	0(0)	0.104
Opportunistic infections, n(%)	2(15.4)	0(0)	0.486
All-cause mortality, n(%)	1(7.7)	0(0)	1.000
Total, n(%)	9(69.2)#	0(0)	0.002

Note: One patient has both COVID-19 rebounded and Pneumocystis jiroveci pneumonia infection. Among the samples in the study, two patients were treated with nematvelitonavir twice for COVID-19 positive. Thus the number of patients in control group and treat group were 13 and 10, respectively.

## Discussion

This Real-world retrospective observational cohort study focused on kidney transplantation patients with COVID-19. Notably, nirmatrelvir-ritonavir showed remarkable efficacy in this patient population, achieving a 100% clinical cure with few adverse reactions. Although tacrolimus was withheld for 12 hours prior to the commencement of nirmatrelvir-ritonavir therapy, tacrolimus concentration remained above 83% of the baseline level during the ritonavir administration. At 48 hours after the cessation of ritonavir, starting tacrolimus at a low dose of 20–25% of the baseline, with daily increments of 20–25% of the baseline dose, allowed a faster and more consistent maintenance of the tacrolimus concentration within the desired range. Remarkably, in the subsequent six - month follow - up period, compared to the control group, patients in the experimental group exhibited significantly lower rates of events such as COVID-19 rebound or delayed conversion to negative, hospitalization due to creatinine elevation, opportunistic infections, and all-cause mortality. The medication strategy proposed in this study required patients to detect blood concentration only once 24 hours after re-starting tacrolimus. Based on whether it was within the target concentration range, patients could determine the precise timing (either on D6 or D7) to restore the original dosage, making the medication process more concise and safe for patients.

Renal transplant patients are at high risk of COVID-19 infection and developing severe disease. Currently, the most effective drugs for Omicron infection mainly include nirmatrelvir-ritonavir, *etc.* Nirmatrelvir is a 3CLpro inhibitor that alleviates disease progression by blocking the replication of coronavirus. It is a substrate of CYP3A, and ritonavir is a potent and irreversible inhibitor of CYP3A4 and also an inhibitor of the P-glycoprotein transporter. Combined use can increase the blood concentration of nirmatrelvir, thereby enhancing its efficacy. Tacrolimus is a substrate of CYP3A4/5 and is transported by P-glycoprotein. When combined with ritonavir, it can lead to a significant increase in concentration, which results in elevated serum creatinine levels, aggravated infections, and even COVID-19 re-positivity, posing a significant threat to patient safety [25]. In our research, we also observed similar clinical phenomenon, as mentioned in the Results section. Some patients experienced a distinct increase in creatinine levels due to they did not follow medical advice and self-administering tacrolimus, which gradually returned to pre-admission levels after the discontinuation of the medication.

Furthermore, although the half-life of ritonavir is approximately 5.6 hours, its inhibitory effects on CYP3A4 and 3A5 persist even after discontinuation. Studies have shown that CYP3A activity reaches nearly 75% of its metabolic activity after 48 hours of ritonavir withheld, and fully recovers at 2–5 days [18,19,26]. Our results demonstrated that 48 hours after ritonavir discontinuation, the tacrolimus concentration, which had been discontinued for seven days, could still reach 60.5% of the baseline concentration. This implies that adding tacrolimus before this time point may lead to a sharp increase in its concentration.

In our study, patients in the experimental group discontinued tacrolimus 12 hours before starting nirmatrelvir-ritonavir therapy, and 48 hours after nirmatrelvir-ritonavir withdrawal, they began administering tacrolimus at 20–25% of the original dose, gradually increasing daily. By the sixth to seventh days after nirmatrelvir-ritonavir discontinuation, all patients had resumed their original tacrolimus dosage. Although the blood concentration of FK506 gradually decreased after tacrolimus was suspended, reaching approximately 60% of the baseline concentration on the seventh day. Due to the temporary discontinuation of the medication, there was no peak concentration, resulting in an even lower AUC for tacrolimus. However, multiple studies have shown that after SARS-CoV-2 invades the human body, it impairs the host immune system in various ways, leading to a reduction or even exhaustion of T cells [27–34]. Consequently, patients experience a weakened immune status during COVID-19 infection, which reduces the risk of acute kidney transplant rejection. Therefore, there is no urgent need to resume tacrolimus therapy prematurely to maintain the pre-infection AUC levels. Furthermore, excessive efforts to achieve baseline tacrolimus concentration levels during this period may hinder virus clearance, delay recovery, and even increase the risk of rebound [35]. Consistent with this, our follow-up data revealed that even when tacrolimus was discontinued during nirmatrelvir-ritonavir therapy and gradually added at low doses, none of the patients experienced acute rejection during the 6-month follow-up period. Conversely, patients who prematurely resumed tacrolimus exhibited delayed COVID-19 negativity or recurrence and an increased risk of opportunistic infections. Nonetheless, for patients with high immunological risk factors, such as those with recent transplants or a history of rejection, clinicians may strongly consider allowing patients to achieve low tacrolimus concentrations on the third day of nirmatrelvir-ritonavir treatment to avoid suboptimal treatment levels. Alternatively, they may opt to restart low-dose tacrolimus immediately after completing nirmatrelvir-ritonavir therapy or adopt a close monitoring and level-based dosing approach [36].

In our experimental group, some patients failed to reach the ideal tacrolimus blood concentration levels. This indicates that our medication strategy has individual differences among patients. One possible cause of this variation is the individual differences in ritonavir metabolism. Due to these differences, in some patients, tacrolimus levels do not quickly reach the target range after restarting according to this regimen. Future studies should consider the genotype distribution of genes related to ritonavir metabolism, such as CYP3A and CYP2D6, to stratify populations and develop personalized dosing regimens. For example, ritonavir fast metabolizers may need to restore their tacrolimus dose more rapidly, while slow metabolizers might require a delayed restoration of their original tacrolimus dose.

This study had some limitations. Firstly, it focuses on patients who had been in a stable condition for one month or more after kidney transplantation. Consequently, the research did not explore how to adjust tacrolimus dosing for patients with high immunological risk factors, such as those who had recently undergone transplantation or had a history of rejection. Secondly, we did not examine the safe administration of tacrolimus in combination with CYP3A enzyme inhibitors, suching as Wuzhi Capsules. Thirdly, this was a single-center small-sample real-world study, and the findings needed further validation through multi-center and expanded samples.

## Conclusion

Our findings present a refined dosing regimen that can mitigate the interaction between nirmatrelvir-ritonavir and tacrolimus. For renal transplant recipients, the judicious use of nirmatrelvir-ritonavir can be of great significant in treating COVID-19 infection more effectively, thereby shortening the disease duration and minimize the occurrence of severe cases. The gradual introduction of tacrolimus with daily increments of 20–25% of the baseline dose, starting 48 hours after the cessation of nirmatrelvir-ritonavir, can reduce the risk of COVID-19 recurrence and opportunistic infections, while maintaining a low risk of allograft rejection. This approach ensures the medication safety for renal transplant patients after COVID-19 infection.

## Supporting information

S1 Appendix Research data. (XLSX)

## Abbreviation:

KTR: Kidney transplantation recipient
COVID-19: Coronavirus disease 2019
TDM: Therapeutic drug monitoring
SOTR: Solid organ transplant recipient
CYP450: Cytochrome P450
SARS-CoV-2: Severe acute respiratory syndrome coronavirus 2
AUC: Area under the curve
CMIA: Chemiluminescent Microparticle Immunoassay
GFR: glomerular filtration rate
CMV: Cytomegalovirus
PJP: Pneumocystis jiroveci pneumonia
3CLpro: 3C-like protease
